# The RITMIA™ Smartphone App for Automated Detection of Atrial Fibrillation: Accuracy in Consecutive Patients Undergoing Elective Electrical Cardioversion

**DOI:** 10.1155/2019/4861951

**Published:** 2019-07-02

**Authors:** Claudio Reverberi, Granit Rabia, Fabrizio De Rosa, Davide Bosi, Andrea Botti, Giorgio Benatti

**Affiliations:** ^1^Gemini Lab, Pl. Badalocchio 3/A, 43126 Parma, Italy; ^2^University Hospital Maggiore, Cardiology Unit, Viale Gramsci 14, 43126 Parma, Italy

## Abstract

**Background:**

The RITMIA™ app (Heart Sentinel™, Parma, Italy) is a novel application that combined with a wearable consumer-grade chest-strap Bluetooth heart rate monitor, provides automated detection of atrial fibrillation (AF), and may be promising for sustainable AF screening programs, since it is known that prolonged monitoring leads to increased AF diagnosis.

**Objective:**

The purpose of this study was to examine whether RITMIA™ could accurately differentiate sinus rhythm (SR) from AF compared with gold-standard physician-interpreted 12-lead electrocardiogram (ECG).

**Design:**

In this observational prospective study consecutive patients presenting for elective cardioversion (ECV) of AF, from November 2017 to November 2018, were enrolled. Patients underwent paired 12-lead ECG and RITMIA™ recording, both before and after ECV procedure. The RITMIA™ automated interpretation was compared with 12-lead ECG interpreted by the agreement of two cardiologists. The latter were blinded to the results of the App automated diagnosis. Feasibility, sensitivity, specificity, and K coefficient for RITMIA™ automated diagnosis were calculated.

**Results:**

A total of 100 consecutive patients were screened and enrolled. Five patients did not undergo ECV due to spontaneous restoration of SR. 95 patients who actually underwent ECV were included in the final analysis. Mean age was 66.2±10.7 years; female patients were 20 (21.1%). There were 190 paired ECGs and RITMIA™ recordings. The RITMIA™ app correctly detected AF with 97% sensitivity, 95.6% specificity, and a K coefficient of 0.93.

**Conclusions:**

The automated RITMIA™ algorithm very accurately differentiated AF from SR before and after elective ECV. The only hardware required by this method is a cheap consumer-grade Bluetooth heart rate monitor of the chest-strap type. This robust and affordable RITMIA™ technology could be used to conduct population-wide screening in patients at risk for silent AF, thanks to the long-term monitoring applicability.

## 1. Introduction

Atrial fibrillation (AF) represents the most common arrhythmia with an estimated prevalence between 2.7 million and 6.1 million American adults, and its prevalence is expected to double over the next 25 years [1]. AF is associated with a 2-fold increased risk for mortality and 5-fold for cerebrovascular events. Unfortunately, it may be an insidious condition, since approximately one-third of patients affected by AF, often paroxysmal, are asymptomatic [2, 3] and a missed AF diagnosis means no access to effective stroke prevention therapies using oral anticoagulation. 

In fact, previous studies have demonstrated that one-third of ischemic strokes are correlated with a history of either previously known or unknown atrial fibrillation and in up to 10% of patients affected by ischemic stroke the arrhythmia is newly diagnosed at the time of hospital admission [4, 5]

The most recent AF European Guidelines recommend opportunistic screening by pulse taking or ECG rhythm strip in individuals older than 65 years of age and advocate for the implementation of new smart technologies for this purpose [6].

Pulse taking is generally limited by its low specificity and by the fact that it is performed by a minority of general practitioners in real world practice, due to time constraints and to the increasing use of automated blood pressure monitors. 12-lead ECG strips, although favoured by higher sensitivity and specificity, are limited by the need of interpretation by qualified specialists and by the “snapshot” nature of rhythm assessment, leading to a high rate of missed-diagnosis in case of paroxysmal and/or silent AF episodes [7].

Repeated Holter monitoring (or use of implantable devices) is not deemed sustainable for systematic screening of AF. Previous studies have demonstrated that there is an incremental diagnostic yield by prolonging monitoring period for the detection of AF, and this holds true up to few years of monitoring duration [8, 9]. New portable technologies have been developed for the automated detection of AF [10–12], showing promising but less-than-ideal diagnostic results.

Some systems use 30-second single-lead ECG data to output an automated rhythm diagnosis (Kardiaband/Apple watch™, for example), but they were demonstrated to be able to interpret, and not always correctly, only approximately 2/3 of rhythm strips [11]. Further, they require active participation of the subject to the acquisition phase, making only snapshots of cardiac rhythm feasible, not allowing for long-term monitoring, which is needed to increase the detection rate of intermittent AF. For heart rate (HR) monitoring some devices use photoplethysmographic sensors, which are too noisy and dependent on body motion for arrhythmia detection, whatever the postprocessing used, since this technology is not able to acquire beat-to-beat intervals reliably. [13, 14]

We aim to test this novel monitoring system combining a consumer-grade Bluetooth low-energy (BLE) HR monitor, of the chest-strap type, and an application for smartphones called RITMIA™ (Heart Sentinel srl, Parma, Italy). This application acquires real-time beat-to-beat RR interval data, sensed by the HR monitor, and continuously applies a patent-pending algorithm able to generate a diagnosis of either probable AF or non-AF rhythm through the entire monitoring period, using a combination of indexes of RR variability and randomness, critically increased in AF.

In the current study we aimed to assess the accuracy of such affordable tool for long-term self- or telemonitoring of cardiac rhythm, to automatically detect the presence of AF on a test bench of 100 consecutive AF patients undergoing elective cardioversion (ECV).

## 2. Materials and Methods

### 2.1. Study Design

The current study is a validation study and has an observational prospective single-center design. To validate the AF detection accuracy of this system, we applied it on a population of unselected ambulatory patients diagnosed with AF and scheduled for elective electrical cardioversion (ECV), using physician-interpreted 12-lead ECG as the reference method, both before and after the ECV procedure.

### 2.2. Monitoring System

The system for automated AF detection tested in the current study integrates two components. The first component is a commercially available BLE HR monitor of the chest-strap type, one among the many HR monitors available on the market able to continuously acquire RR interval data [13] from the electric cardiac signal sensed through two integrated electrodes in stable contact with the skin. The integrated electronic device filters out noncardiac electrical signals and lower-amplitude nonventricular signals, such as atrial activity waves, sending only R-R interval data (and averaged HR) through wireless BLE standard protocol. The second component of the system is the new application for smartphones in test (available only for research) called RITMIA™ (Heart Sentinel srl, Parma, Italy), run on either iOS (version 10-12) or Android (version 7-8) smartphones in the current study.

The application receives the RR interval data, as a measure in milliseconds and, by the continuous computation of its proprietary algorithm (patent-pending), generates a real-time rhythm diagnosis (“probable AF,” “normal rhythm,” or “unclassified non-AF arrhythmia”) using a data matrix comprising a number of immediately prior R-R intervals; the full RR dataset is uploaded via the internet in near real-time to a password-protected cloud server, which can be accessed for revision only by an authorized physician. This also allows for the confirmation and quantification of the percentage AF burden along the entire monitoring period. The patent-pending algorithm is based on a weighted combination assessment of RR interval variability and randomness/chaos, an index which finally measures RR heterogeneity [15]. The RITMIA™ app automatically labels each RR interval in a color-coded fashion, based on the derived index from a number of immediately prior RR intervals, into three possible classes: “probable atrial fibrillation” (red color-coded), “normal rhythm” (blue color-coded), or “unclassified non-AF arrhythmia” (yellow color-coded), which comprehends any type of rhythm or arrhythmia that falls between the established cutoffs of the two preceding rhythms, but still represents non-AF rhythm for the sake of the final interpretation.

### 2.3. Study Population

**Figure 1:**
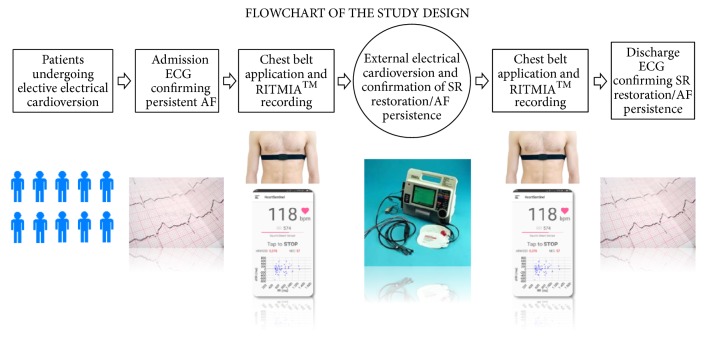
Flowchart of the study design. Figure also shows system components and in particular an example of a chest-belt HR monitor sensor similar to the ones used in the study and an example of RITMIA™ App interface. AF: atrial fibrillation, SR: sinus rhythm.

One hundred consecutive patients undergoing elective ECV from November 2017 to November 2018, because of AF, according to the inclusion and exclusion criteria, were enrolled in the study. On the day of the ECV procedure, all patients underwent a 12-lead ECG immediately followed by the application of the RITMIA™ system for at least 10 consecutive minutes, immediately before and after the ECV procedure. In conclusion, two couples of 12-ECG snapshot tracing + RITMIA™ 10-minute recordings (before and after ECV) were obtained for all patients. (Figure 1)

### 2.4. Inclusion Criteria

- Age > 18 years
- Patients affected by atrial fibrillation (AF) undergoing elective external electrical cardioversion (ECV)
- CHA2DS-VASC score≥2
- Signed informed consent`

### 2.5. Exclusion Criteria

(i) Presence of pacemaker or automatic internal cardioverter defibrillator.

Every patient was monitored with a personal chest belt HR sensor, connected via BLE to a dedicated smartphone running the RITMIA™app, that was kept by one of the investigators in the same room for the entire duration of the recordings. (Figure 1)

12-lead ECGs were interpreted at the moment of acquisition upon agreement of two cardiologists (delegates of the PI). On the contrary, the data collected by the chest belt HR sensor were analysed in real-time by the algorithm of the RITMIA™ app and directly uploaded and collected for review in the cloud-based server.

RITMIA™ 10-min recordings with full RR interval data (and the labelling of rhythm) were accessible only by the investigators or authorized delegates. The two cardiologists who interpreted 12-lead ECGs were blinded to the results produced by the RITMIA™ app.

**Figure 2:**
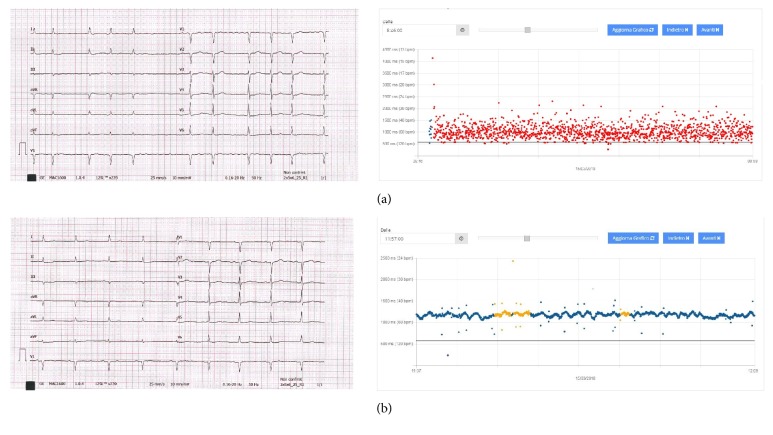
Examples of 12-lead ECG (left side) with the cloud-based interface of the RITMIA™ App showing the paired recording (right side). RR intervals are plotted on a graph showing RR interval on the y-axis and time on the x-axis. A colour-code is automatically attributed to each dot, corresponding to the automated diagnosis as “probable AF”=red, “unclassified non-AF arrhythmia”=yellow and “normal rhythm”=blue. (a) pre-ECV 12-lead ECG showing atrial fibrillation and paired App recording automatically labelled as “probable AF”. (b) post-ECV 12-lead ECG showing restoration of sinus rhythm and paired App recording automatically labelled as “normal rhythm” and “unclassified non-AF arrhythmia”. ECV: electrical cardioversion. AF: atrial fibrillation.

As described above, the automated algorithm classifies each acquired beat as “probable AF,” “unclassified non-AF arrhythmia,” or “normal rhythm” and updates the diagnosis second by second. The result is a map of coloured dots plotted on a graph that display time on the x-axis and RR interval (HR) on the y-axis (Figure 2).

The study protocol conforms to the ethical guidelines of the 1975 Declaration of Helsinki and was approved by the institution's human research committee.

## 3. Statistical Analysis

Continuous variables are expressed as mean (standard deviation, SD) and categorical variables as counts (percentage), as appropriate.

In order to determine the diagnostic performance of the new system, the automated detection of the RITMIA™ app was compared with the paired physician-interpreted 12-lead ECGs by calculating the sensitivity, specificity, and *ƙ* coefficient for intermethod agreement.

RITMIA™ “probable AF” arrhythmia classification matching AF at 12-lead ECG was considered a true positive, while RITMIA™ showing “normal rhythm” or “unclassified non-AF arrhythmia” with corresponding sinus rhythm or any other type of arrhythmia on 12-lead ECG was a true negative.

*ƙ* coefficients greater than 0.80 were considered indicative of optimal agreement.

For all interpretations and statistical analysis, each 10-min recording was considered diagnostic of the presence of AF if the rhythm had been automatically labelled as “probable AF” (red-color labelling) for at least 90% of the total duration of the recording, in order to be easily compared in a binary Yes/No fashion with the standard 12-lead ECG, which instead shows only few seconds of cardiac activity.

## 4. Results

We screened and enrolled a total of 100 patients between November 2017 and November 2018, of which 5 were excluded due to the documentation of spontaneous restoration of normal sinus rhythm the day before the procedure. Mean age was 66.2±10.7 years and 21% of the patients were women (n=20). Study population characteristics are summarized in Table 1. All patients were able to complete the protocol with no complaints of discomfort while wearing the chest-belt HR sensor. For each patient a 12-lead ECG and RITMIA™ 10-min recording was acquired before and after the procedure. A total of 95 patients underwent ECV, of whom 87 (92%) were with a diagnosis of persistent AF and 8 (8%) of persistent atrial flutter (AFL). The procedure was effective, with restoration of normal sinus rhythm, in 83 (87%) of the 95 patients.

Automated diagnosis of the n=190 RITMIA™ 10-min recordings obtained was compared with corresponding physician-interpreted 12-lead ECGs (Table 2).

The recordings of the 8 patients unexpectedly found with AFL at the moment of their ECV (AF had been diagnosed at prior clinical contact) were excluded from the AF accuracy metrics (see Tables 1 and 2), and AFL was rather considered a standalone category, although such tracings were correctly classified as non-AF arrhythmia by the RITMIA™ automated diagnosis. Resulting AF prevalence among the residual 182 ECG tracings (190 minus the 8 AFL) was consequently 54.4%.

The ECG tracings demonstrating sinus rhythm (with or without premature beats) were correctly labelled by the application as “normal rhythm” or “unclassified non-AF arrhythmia,” except for 4 false positives (i.e., RITMIA™ automated diagnosis was ”probable AF”), in patients with successful restoration of sinus rhythm, all demonstrating very frequent supraventricular ectopic beats, as it sometimes happens after ECV. In conclusion 79 out of 83 ECG with sinus rhythm were correctly classified as sinus rhythm or non-AF arrhythmia (true negatives=79, false positives=4).

The ECG tracings demonstrating AF were correctly labelled by the application as “probable AF,” except for 3 false negatives (i.e., output was ”unclassified non-AF arrhythmia”). In conclusion, 96 out of 99 ECG with AF were correctly classified as “probable AF arrhythmia” (true positives=96, false negatives=3) by RITMIA™.

The RITMIA™ system performance in terms of automated detection of AF in comparison with 12-lead ECG showed sensititvity, specificity, and kappa coefficient values of, respectively, 97.0% (95% CI 91.4-99.4%), 95.2% (95% CI 89.1-98.8%), and 92.6% (95% CI 87.3-98.0%).

## 5. Discussion

Atrial fibrillation represents a great concern for public health for mainly two reasons: it is a condition associated with a significantly higher risk of morbidity and mortality and its prevalence is increasing due to the aging population.

As stated above, the disease is insidious with an asymptomatic course in up to one-third of patients, the so-called silent atrial fibrillation [2, 3], posing these patients to an avoidable risk of thromboembolic events.

Given the magnitude of the problem, in the recent years many international societies have focused on the topic, calling for the need to gather more evidence-based efficacy and safety data and implement new technologies for AF screening [16–18].

New, noninvasive technologies are flourishing and many new devices (e.g., modified blood pressure monitors and photoplethysmography-based (PPG) or single-lead ECG-based devices) have been tested in the recent years, showing promising results. However they have some key limitations, in view of wide population applicability: firstly, all of them require a specific-branded and costly hardware device; secondly, almost all of them need an active participation of the patient to the recording phase, raising doubts on their utility for the detection of asymptomatic episodes in the long term; thirdly, they acquire a limited time-frame recording, limiting their capability to detect paroxysmal events.

The present validation study compared the diagnostic accuracy of a new system composed of a nonproprietary commercially available HR chest-belt sensor and an application for smartphones versus physician-interpreted 12-lead ECG, for the diagnosis of AF in a population of patients undergoing elective ECV.

The experimental system automated diagnosis was successfully perfomed in all patients, with no need to exclude any patient from the analysis, and it recognized “probable AF” rhythm with 97% sensitivity and 95.2% specificity.

This high sensitivity value is in line with other screening tests for the detection of AF, as demonstrated by a 2017 review of 15 studies [7]. That review paper also demonstrated lower mean specificity values for PPG systems (86%) and comparable mean specificity values for single-lead ECG systems (94%).

Furthermore, our results are in agreement with a recent study by Lown et al., which showed comparable accuracy data for AF detection of a system based on a consumer chest-belt HR sensor (96.3% sensitivity and 98.2% specificity), similar to the one used in our study, and other “smart” devices such as Kardia AliveCor™ (87.8% sensitivity and 98.8% specificity). Interestingly, this study also confirmed the full first-attempt readability of RR data obtained with chest-belt sensors (as opposed, for example, to 15.5% of patients requiring more than one attempt with Kardia AliveCor™) [19].

Only another study that tested AF detection accuracy of a single-lead ECG-based tool was conducted on a population of patients with a known history of AF undergoing elective ECV [11]. In this study, however, 20% of recordings obtained with the single-lead ECG wrist-band device were labelled as “unclassified” either due to artifacts, low-amplitude signals, or unclear reasons (of these, 13% remained unclassified even after revision of an elechtrophysiologist). Among the classifiable tracings, the automated algorithm detected AF with 93% sensitivity, 84% specificity, and 0.77 k coefficient, all values that are clearly inferior to the RITMIA™ system tested in the current study.

The system we tested presents several advantages for the use in the prolonged monitoring/screening in search of AF. It can be very easily applied or removed by patients at their best ease or in case of symptoms: this intermittent monitoring, smartly self-operated (the RITMIA™ app runs in background mode on the smartphone and monitoring starts and stops automatically when the chest strap is weared or removed), can possibly increase subjects' compliance and lead to potentially unlimited rhythm data collection. It does not require an active participation by the subject, apart from the wearing phase, as it would happen with systems acquiring 30-second ECG snaphots; those systems are useful for automatic diagnostic interpretation of an ECG, but not for prolonged rhythm monitoring. Moreover, the only hardware required in the system is inexpensive (chest-strap HR monitors with RR capability are priced in the range of 20-80 dollars depending on specific vendor) and relies on very robust time markers from ventricular electrical activity (i.e., R-R- intervals), unaffected by body motion.

There are also some cons. The system is based on the analysis of RR heterogeneity, precluding the option of physicians' overreading for direct ECG confirmation of AF. Therefore, every positive result would need further diagnostic ECG testing, but this holds true for all non-ECG screening devices. Furthermore, as the prevalence of AF in the study population (54.4%) is much higher than the estimated prevalence of undetected AF in the community (0.4% overall and 1.4% for individuals of >65 years of age) [1], the accuracy of the experimental system may be different when applied for screening purposes to the general population.

As proven in the present study, the system is unable to specifically classify, if not generically as “unclassified non-AF arrhythmias,” regularly irregular rhythms, such as atrial flutter and presumably atrial tachycardias with fairly regular ventricular response. Lastly, a chest strap may be perceived as annoying in daily life, although wrist PPG devices may not be less annoying, since they need to be very tightly weared to be able to work decently, and also after this operation, they remain insufficiently accurate for arrhythmia detection.

This diagnostic accuracy comparative study was conducted on a population of patients with a known history of atrial fibrillation. Future research is needed to determine the performance of the system on the detection of new-onset atrial fibrillation and on a target age-range population, such as individuals older than 65 years of age.

## 6. Conclusions

A novel system composed of an inexpensive consumer-available chest-belt HR sensor and an application for smartphones (RITMIA™) very accurately differentiated AF arrhythmia from non-AF rhythm in a cohort of ambulatory patients undergoing elective ECV. This robust, affordable, and accurate RITMIA™ technology could be tested in the future to conduct wide-population screening in patients simply at risk for silent AF, thanks to the long-term and cost-effective monitoring applicability.

## Figures and Tables

**Table 1:** Study Population Characteristics.

STUDY POPULATION CHARACTERISTICS (N=95)	
Age (years)	66.2±10.7
Male/Female	75/20
AF	87 (91.6%)
AFL	8 (8.4%)
CHA2DS2-VASC score	2.3±1.5
Preprocedural TEE	59 (62.1%)

ANTICOAGULANT	

Warfarin	32 (33.7%)
Dabigatran	18 (18.9%)
Rivaroxaban	22 (23.2%)
Apixaban	20 (21.1%)
Edoxaban	3 (3.2%)

Successful ECV	83 (87.4%)

Study population characteristics. AF=atrial fibrillation; AFL=atrial flutter; TEE=transesophageal echocardiography; ECV=electrical cardioversion.

**Table 2:** Ritmia Automated Diagnosis Compared To 12-Lead ECG Diagnosis.

12-Lead ECG	APP AF	APP NO-AF	Total
AF	96	3	99
Flutter	0	8	8
SR	4	79	83
Total	100	90	*TOT=190*

Contingency table showing comparison between 12-lead ECG diagnosis and RITMIA™ automated diagnosis.

## Data Availability

The data used to support the findings of this study are available from the corresponding author upon request.
